# Accuracy of a Commercial Large Language Model (ChatGPT) to Perform Disaster Triage of Simulated Patients Using the Simple Triage and Rapid Treatment (START) Protocol: Gage Repeatability and Reproducibility Study

**DOI:** 10.2196/55648

**Published:** 2024-09-30

**Authors:** Jeffrey Micheal Franc, Attila Julius Hertelendy, Lenard Cheng, Ryan Hata, Manuela Verde

**Affiliations:** 1 Department of Emergency Medicine University of Alberta Edmonton, AB Canada; 2 CRIMEDIM—Center for Research and Training in Disaster Medicine, Humanitarian Aid and Global Health Universita' del Piemonte Orientale Novara Italy; 3 Department of Information Systems and Business Analytics College of Business Florida International University Miami, FL United States; 4 Department of Emergency Medicine Beth Isreal Deaconess Medical Center Harvard Medical School Teaching Hospital Boston, MA United States; 5 Emergency Medicine Department National University Hospital National University Health System Singapore Singapore; 6 Department of Emergency Medicine Indiana University School of Medicine Indianapolis, IN United States

**Keywords:** disaster medicine, large language models, triage, disaster, emergency, disasters, emergencies, LLM, LLMs, GPT, ChatGPT, language model, language models, NLP, natural language processing, artificial intelligence, repeatability, reproducibility, accuracy, accurate, reproducible, repeatable

## Abstract

**Background:**

The release of ChatGPT (OpenAI) in November 2022 drastically reduced the barrier to using artificial intelligence by allowing a simple web-based text interface to a large language model (LLM). One use case where ChatGPT could be useful is in triaging patients at the site of a disaster using the Simple Triage and Rapid Treatment (START) protocol. However, LLMs experience several common errors including hallucinations (also called confabulations) and prompt dependency.

**Objective:**

This study addresses the research problem: “Can ChatGPT adequately triage simulated disaster patients using the START protocol?” by measuring three outcomes: repeatability, reproducibility, and accuracy.

**Methods:**

Nine prompts were developed by 5 disaster medicine physicians. A Python script queried ChatGPT Version 4 for each prompt combined with 391 validated simulated patient vignettes. Ten repetitions of each combination were performed for a total of 35,190 simulated triages. A reference standard START triage code for each simulated case was assigned by 2 disaster medicine specialists (JMF and MV), with a third specialist (LC) added if the first two did not agree. Results were evaluated using a gage repeatability and reproducibility study (gage R and R). Repeatability was defined as variation due to repeated use of the same prompt. Reproducibility was defined as variation due to the use of different prompts on the same patient vignette. Accuracy was defined as agreement with the reference standard.

**Results:**

Although 35,102 (99.7%) queries returned a valid START score, there was considerable variability. Repeatability (use of the same prompt repeatedly) was 14% of the overall variation. Reproducibility (use of different prompts) was 4.1% of the overall variation. The accuracy of ChatGPT for START was 63.9% with a 32.9% overtriage rate and a 3.1% undertriage rate. Accuracy varied by prompt with a maximum of 71.8% and a minimum of 46.7%.

**Conclusions:**

This study indicates that ChatGPT version 4 is insufficient to triage simulated disaster patients via the START protocol. It demonstrated suboptimal repeatability and reproducibility. The overall accuracy of triage was only 63.9%. Health care professionals are advised to exercise caution while using commercial LLMs for vital medical determinations, given that these tools may commonly produce inaccurate data, colloquially referred to as hallucinations or confabulations. Artificial intelligence–guided tools should undergo rigorous statistical evaluation—using methods such as gage R and R—before implementation into clinical settings.

## Introduction

Despite the progression of computerized artificial intelligence (AI) since the 1950s, its use has largely remained unreachable for clinicians. Recently, chat-oriented large language models (LLMs) have presented a markedly more user-friendly alternative. Engaging with these models involves a straightforward, conversational interaction: the user provides text input (referred to as the prompt), and the model responds with text output. The public launch of ChatGPT (OpenAI) in November 2022 significantly lowered the barriers to AI use by offering access to an LLM via an uncomplicated web interface, thereby enabling anyone with fundamental computer knowledge to leverage AI for any issue that can be articulated in text. The success of an innovation, such as ChatGPT, depends on its use by end users, which requires acceptability, acceptance, and adoption which is often complex when applied in the clinical setting [1].

Although chat-based LLMs are powerful and intuitive, they are not without shortcomings and have two major faults: (1) hallucinations or confabulations: LLMs can generate outputs, or “hallucinations,” that are syntactically correct and superficially plausible but factually inaccurate or entirely fabricated [2]. These issues arise due to the model’s lack of a grounded understanding of reality and its reliance on patterns in the training data. For instance, it might generate dates, events, or statements that are completely false but sound believable. Addressing hallucinations is a significant challenge, and researchers are exploring various techniques, including refining training processes and developing mechanisms to verify generated information against reliable sources [3]. (2) Prompt dependence and promptology: the way prompts are framed can significantly influence the responses generated by LLMs [4]. Slight changes in phrasing or specifying different contexts can yield varying outputs. While it allows for some level of “tuning” of the responses by modifying the prompt, it also introduces challenges related to the consistency and reliability of the outputs. The study and practice of crafting and optimizing prompts to extract desired outputs from LLMs is sometimes referred to as “prompt engineering” or “promptology.” It has become a subfield of its own, where researchers explore how to guide the models efficiently to produce useful and reliable responses.

These limitations underscore the importance of continued research and development in the field of AI and natural language processing. To make these models more reliable, accountable, and useful, researchers and engineers use various strategies such as integrating external knowledge bases for fact-checking, improving the training data and methodology, and developing ways to mitigate biases present in the outputs. The ongoing research and development aim to improve the robustness, reliability, and utility of LLMs while also ensuring that they are used ethically and responsibly across various applications.

Among the many possible uses for ChatGPT in medicine, patient triage is an interesting case study. AI-guided triage could be particularly useful in disaster medicine, where circumstances may require inexperienced health care providers to perform triage. One can imagine triage performed by AI where the provider enters patient information as text, and the patient’s triage code is outputted. This might allow physicians, first responders, nurses, or even the patients themselves to perform triage. However, at present, it is unclear whether LLMs can provide sufficiently accurate triage for use in a disaster.

Presently, the Simple Triage and Rapid Treatment (START) algorithm, which divides disaster casualties into four different triage codes, is the most commonly used model for disaster triage [5]. Disaster casualties are classified into four groups that are named by priority and color: red (immediate), yellow (delayed), green (ambulatory), and black (expectant) [5]. This model is based on a simple flowchart and seems ideal for use by AI models.

To date, there is a paucity of studies that examine the use of ChatGPT among health care providers expected to perform triage in a disaster setting. Our study extends the work by Liu et al [6] that examined the AI-generated suggestions from ChatGPT to optimize clinical decision support. Additionally, our research extends a call for additional research that examines AI chatbot efficacy by evaluating the precision of different AI algorithms in conducting disaster triage [7].

Furthermore, there is a growing body of literature that examines the factors that influence the acceptability of and adoption of ChatGPT [8]. The extant literature provides insights into health domains and common medical pathways where chatbots have been studied.

This study addresses the research problem: “Can ChatGPT adequately triage simulated disaster patients using the START protocol?” This study used ChatGPT to triage simulated patient vignettes and assessed three major outcomes:

- Repeatability: variation in response to repeated use of the same prompt with the same patient vignette.
- Reproducibility: variation in response with the use of different prompts with the same patient vignette.
- Diagnostic accuracy: overall accuracy of triage when compared to a documented reference standard.

## Methods

### Study Design

This study was based on a crossed gage repeatability and reproducibility (gage R and R) study with a comparison to a standard [9]. An easily approachable summary of this methodology is available on the American Society of Quality website [10].

The gage R and R study was chosen as the preferred methodology for several reasons. First, it is well documented in the industrial quality control literature as a valid and reliable means to measure the accuracy of instruments by ensuring results are repeatable and reliable [11]. Second, the method can separate variation due to the tool (ChatGPT) and variation due to the operators (prompts). Third, the method allows the calculation of repeatability and reproducibility without reliance on a reference standard. Fourth, the method is based on the calculation of the range: START scores are ordinal (not categorical or ratio) and should be analyzed with methods that do not rely on linear or normal data. Finally, the use of this well-documented method allows other researchers to reproduce the study.

Gage R and R studies use repeated measurements of multiple parts by various appraisers using the same equipment. This ensures that the output is the same as the input and that the same measurements occur over time [11]. In this study, the patient vignettes are the different “parts” while the various prompts are the “appraisers.” Overall variability is broken down into repeatability (variation when the same prompt is used repeatedly on the same vignette) and reproducibility (variation when different prompts are used on the same vignette). In this study, 9 prompts (appraisers), 391 simulated patients (parts), and 10 repetitions of each combination were performed. In total, there were 3519 unique combinations of prompt and vignette, and a total of 35,190 simulated patient triages.

The gage R and R was accomplished in four parts: (1) each combination of prompt and vignette was submitted to ChatGPT ten times via the application programming interface (API). (2) Repeatability was calculated based on the average range of all trials using the same prompt and vignette. In an ideal system—where ChatGPT returns the same response each time—the range would be zero. (3) Reproducibility was calculated based on the average range of all trials using the same vignette. Again, in an ideal system, ChatGPT would give the same result for each vignette regardless of the prompt and the range would be zero. (4) Overall variability due to repeatability and reproducibility (overall gage R and R) was calculated based on the sum of the squares of variation due to repeatability and variation due to reproducibility [11].

### Study Setting

The study was performed in September 2023 using the ChatGPT API version GPT-4.

### Participants

The simulated patient vignettes were based on 391 simulated adult and pediatric patients previously used in disaster medicine training and simulation. A total of 380 simulated patients were taken from the most current version of the Disastermed.ca database which has expanded from its originally published size [12]. As this training set contained few patients with the “Black” triage code, 11 further cases with the published triage standard of “Black” were taken from various previously published training sets [13-17]. The final patient data set of 391 patients contained patients of various ages and diverse traumatic and nontraumatic presentations. Several sample vignettes, in the format that they were used to prompt ChatGPT, are presented in Multimedia Appendix 1. The average age for the simulated patients was 29.9 (SD 18.2; range 0-80) years with 109 (27.9%) patients less than or equal to 16 years of age. Sex was male in 277 (71%) patients and female in 114 (29%) patients.

Reference standards for START were assigned by the research team. First, two authors (JMF and MV) assigned triage codes independently to each simulated patient vignette using the standard START procedure—categorizing each patient as red, yellow, green, or black. Interrater reliability was calculated using Cohen κ [18]. Where the two authors did not agree, a third author (LC) again repeated the triage independently as a tiebreaker, and the triage code assigned by two of the three reviewers was used. All three authors assessing the reference standard triage code had fellowship training or master’s degrees in disaster medicine and between 2 and 15 years of disaster medicine experience.

To preserve the ordinality of the START levels, the assigned triage was rescored as an integer: 1 for red, 2 for yellow, 3 for green, and 4 for black. This order parallels the order of priority given by the North Atlantic Treaty Organization triage system [17].

Patient vignettes were collated into a MySQL (version 8.0.27; Oracle) database.

### Interventions

A total of 9 unique ChatGPT prompts were tested (Multimedia Appendix 2). Each prompt was created by one of the study authors. Authors were asked to create their prompts independently without consultation with the other authors.

### Outcome Measures

Three outcome measures were determined: (1) repeatability (equipment variation): the extent to which repeated use of the same prompt for the same patient vignette led to variation in response was measured as a percentage of total variation calculated by the range method [11]. This represented variability inherent to the ChatGPT tool itself. (2) Reproducibility (appraiser variation [AV]): the extent to which the use of different prompts on the same patient vignette led to variation in response was measured as a percentage of total variation calculated by the range method [11]. This represented variability due to the use of different prompts. (3) Diagnostic accuracy: agreement of the triage scores was recorded when the ChatGPT gave the same triage code as the reference standard determined by the experts. Overtriage occurred when ChatGPT gave a lower number (higher acuity) rating than the reference standard. Undertriage occurred when ChatGPT gave a higher number (lower acuity) than the reference standard. Accuracy was calculated as a percentage of the total ChatGPT queries that gave a valid response.

Gage R and R was calculated as the summed square of repeatability and reproducibility [11]. Previously published guidelines rate the usability of a measurement tool in relation to the total gage R and R: less than 10% is considered adequate for general use, 10% to 30% adequate for use in low-risk situations, and more than 30% inadequate [9,11].

### Data Collection

The ChatGPT API was queried by an automated Python (version 3.6.9) script (Multimedia Appendix 3) [19].

Briefly, the script looped through each of the nine prompts. Each prompt was combined with each vignette and the text “Answer only with the color code and no other text” was appended. The ChatGPT API was then queried 10 times with this combination, before moving on to the next simulated patient, and finally moving on to the next prompt. In the end, 10 trials of each of the nine prompts were combined with 391 simulated cases for a total of 35,190 simulated patient triages.

Triage scores were translated from a color code to an integer by the script as described in the section “Participants” above.

### Data Analysis

Statistical analysis was performed using Stat59 (STAT59 Services Ltd). As the final triage scores were ordinal, repeatability and reproducibility were calculated with the range method, as this method does not require assumptions of normality or linearity [11].

### Sample Size

No formal sample size or power calculation is available for gage R and R studies. Small sample sizes are customary with gage R and R. The use of 10 parts, 3 operators, and 2 replicates is a common design [10]. This study used a convenience sample of all available simulated trauma casualties from the Disastermed.ca database resulting in a much larger design of 391 parts (vignettes), 9 operators (prompts), and 10 replicates.

### Ethical Considerations

This study involved no human participants. The simulated casualty vignettes were previously written by the first author (JMF) for a simulation exercise [12]. The reference standards for the simulated casualties were assigned by 3 of the study authors (JMF, MV, and LC).

## Results

When the first two raters assigned the START scores to the 391 simulated patients, they agreed on 345 (88.2%). κ for the interrater reliability was 0.81 (95% CI 0.76-0.86). Following the assignment by a third rater to the 46 cases where the initial two raters did not agree, the final data set included 25 simulated patients with the standard values of black, 35 red, 130 yellow, and 201 green.

In total, 35,190 requests were made to the ChatGPT API by the Python script. In 35,102 queries (99.7%), a valid response was returned that contained only the triage color code. In 88 (0.3%) cases, the response returned was not valid: containing either no text or text without a single triage color code. There were 391 unique combinations of prompt and vignette. A frequency table for the range of the 10 trials for each combination is shown in Table 1. Notably, in only 1735 (49.3%) was the range zero: indicating that ChatGPT chose the same triage code for all ten trials. Conversely, in slightly more than half of the cases, each of the ten trials did not give the same response. Repeatability, as calculated by the range method, was 0.17, indicating that 14% of the overall variation was due to equipment variability: variability due to ChatGPT itself.

Table 2 shows the average range for each of the 9 prompts. The lowest average range (mean 0.36, SD 0.59) was for prompt #8, which indicates that this prompt had the best reproducibility. Conversely, the highest average range was for prompt #4 (mean 1.27, SD 0.80) indicating the lowest reproducibility. Reproducibility, as calculated by the range method, was 0.091, indicating that 4.2% of the overall variability was due to AV: variability due to differences in the prompts.

A frequency table of the range for each of the 391 simulated patient vignettes is shown in Table 3. In only 27 (6.9%) was the range zero, indicating that ChatGPT gave the same triage code for all trials and all prompts. The remainder of the variability as calculated by the range method (81%) was due to variation within the case vignettes.

Overall gage repeatability and reproducibility as calculated by the range method was 0.19%.

Table 4 shows the triage accuracy for the nine prompts. The overall accuracy for ChatGPT to perform START was 63.9% (SD 0.25%). Among the nine prompts, the maximum accuracy was 71.8% (SD 0.7%) and the minimum was 46.7% (SD 0.8%).

**Figure 1:**
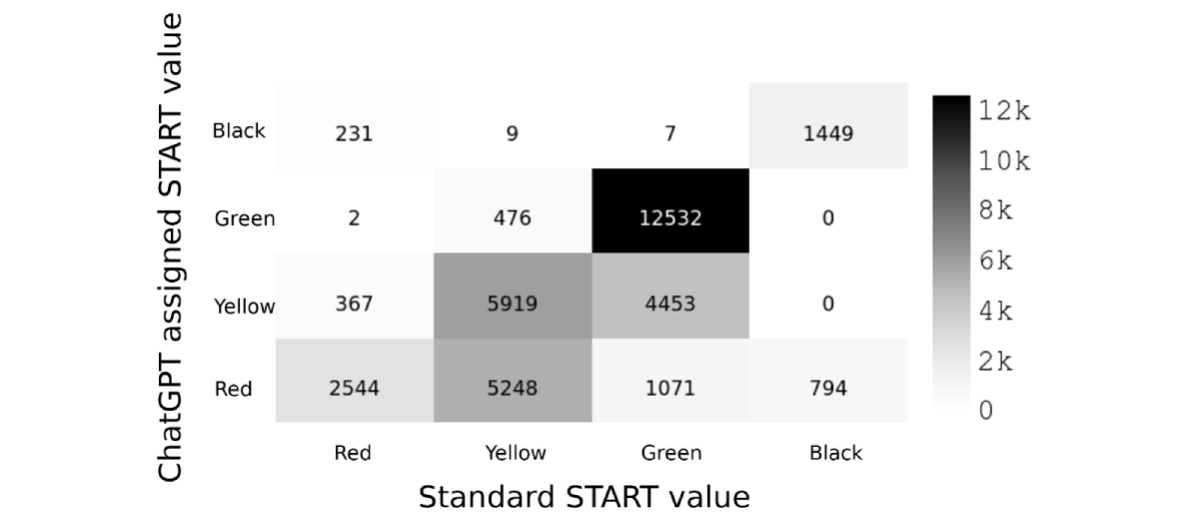
Confusion matrix comparing the ChatGPT assigned START score versus the reference standard. START: Simple Triage and Rapid Treatment.

A confusion matrix of the ChatGPT assigned values of START versus the reference standard is presented in Figure 1. The overtriage rate for all vignettes was 32.9%, with a 3.1% undertriage rate. For the reference standard of red, 80.9% of vignettes matched the reference standard, with a 19.1% undertriage rate. Reference standard yellow patients were triaged matching the reference standard in 50.8% of cases, with a 45% overtriage and a 4.2% undertriage rate. For patients with a reference standard of green, the rate of agreement with the reference standard was 69.4%, with a rate of 30.6% overtriage, and 0.04% undertriage. For patients with a START standard of black, the rate of triage matching the reference standard was 64.6%, with a 35.4% overtriage rate.

**Table 1:** Range of the 10 trials for each of the 3519 unique combinations of prompt and vignette.

Range	Number of observations, n	Percentage of observations, %
0	1735	49.3
1.0	1459	41.5
2.0	226	6.4
3.0	99	2.8

**Table 2:** The average range of the 10 trials for each of the 9 prompts.

Prompt	Range, mean (SD)
1	0.46 (0.66)
2	0.49 (0.7)
3	0.72 (0.68)
4	1.27 (0.80)
5	0.66 (0.76)
6	0.53 (0.61)
7	0.54 (0.62)
8	0.36 (0.59)
9	0.61 (0.72)

**Table 3:** Range of the 10 trials for each of the 391 simulated patients.

Range	Number of observations, n	Percentage of observations, %
0	27	6.9
1.0	174	44.5
2.0	150	38.3
3.0	40	10.2

**Table 4:** Accuracy for each of the 9 prompts.

Prompt	Accuracy (%), mean (SD)
1	71.8 (0.7)
2	72.0 (0.7)
3	63.1 (0.8)
4	46.7 (0.8)
5	68.7 (0.7)
6	60.6 (0.8)
7	66.8 (0.8)
8	54.4 (0.8)
9	71.3 (0.7)

## Discussion

### Interpretation

This study suggests that ChatGPT version 4.0 is inadequate to accurately triage simulated patients using the START model.

Repeatability, variation due to ChatGPT itself, was 14% of the total variation. This indicates excess variability when the same prompt is used multiple times to triage the same patient vignette. Practitioners should be aware that using the same prompt and the same patient data multiple times does not always give the same response.

Reproducibility, or AV, was lower at 4.2% of overall variation, suggesting that the influence of how the prompt is written has less of an effect on total variation than does the inherent variation within ChatGPT itself. Among the prompts, prompt #4 had the most variability, while prompt #8 had the least.

The overall accuracy of ChatGPT (63.9%) to triage patients using START should not be surprising based on how LLMs are developed. LLMs are not expert systems: the software itself has no actual knowledge of the problem it is addressing. The system does not inherently use the same algorithm as a human would to triage patients. Instead, LLMs rely on probability principles that use complex mathematical models to predict text output based on the prompt without any knowledge of the question asked.

### Previous Studies

Previous studies have documented that ChatGPT accuracy and reliability are highly variable [20].

In this study, ChatGPT displayed a lower triage accuracy than reports using traditional human-applied triage. For example, a meta-analysis in 2022 showed the START accuracy of human participants to be 73%, with an overtriage rate of 14% and an undertriage rate of 10% [5]. A recently published study assessed the use of ChatGPT version 3.5 to triage patients using the Canadian Triage and Acuity Score. The study showed similar results with a variation due to reliability of 14%, a variation due to reproducibility of 4.1%, and overall accuracy of 61.4% [21].

### Strengths and Limitations

This study was limited to a single LLM: ChatGPT version 4.0. Accuracy may improve as newer models are made public.

This study was limited to only 9 different prompts. It is possible that further prompt optimization may improve accuracy. Nonetheless, of the 9 prompts, none displayed accuracy adequate to be used for patient care. As these prompts were simply a convenience sample from emergency physicians, the study lacks the robustness to make statistically valid conclusions about the influence of the prompts.

As the primary objective of this study was to assess the performance of the “out of the box” ChatGPT 4, defaults for parameters, such as Temperature and Top_P, were used. It is possible that manipulation of these parameters could improve performance. Furthermore, ChatGPT is a general-purpose LLM, that was not specifically fine-tuned for START. It is possible that fine-tuning of a LLM could lead to improved performance.

The reference standard for the simulated patients relied on expert opinion. Although the experts had extensive training in disaster medicine, in this study experts did not agree 100% on all patients. Thus, triage accuracy recorded in this study should be considered in this context. Nonetheless, it is important to remember that gage R and R studies do not rely on a reference standard. Since we can see that ChatGPT does not answer consistently when given the same question, we know that for certain it cannot always be correct. Furthermore, the simulation scenarios may not be representative of real-world data.

### Significance

#### Clinical Implications

LLMs have been studied for many clinical applications including ophthalmic diagnosis, interpretation of laboratory results, decision support for cardiac diagnoses, and others [22-24]. However, while these models can be valuable, they do have limits to their performance. This study illustrates several commonly encountered errors.

First, this study demonstrates that ChatGPT frequently returns incorrect answers. These are often called hallucinations or confabulations, where ChatGPT returns text that is syntactically correct, but false [25]. In this study, 99.7% of queries gave a response, but only 63.9% were correct. Notably, LLMs rarely indicate if the response is speculative or uncertain, but rather return a single response as if it were a truth. Clinicians should be cautious when using LLMs and be alert to the possibility of hallucinations.

In this study, overtriage (labeling casualties as more acute than the reference standard) was present in 32.9% while undertriage was much lower at 3.1%. An ideal triage system should guard against both errors. For the individual casualty, overtriage is much less dangerous, as it would result in care being given more urgently than needed. Conversely, overtriage, while of no danger to the individual casualty, leads to the risk of overwhelming the medical system.

Toxicity and stereotyping represent other prevalent errors, even though they were not directly examined in this study [25]. Clinicians need to be cognizant of the occurrence of these issues. Enhanced safeguards in recent LLMs are designed to mitigate the frequency of these errors.

LLMs undergo training using text, and some may use user-inputted text for additional training. It is imperative for practitioners to meticulously review the end user agreement of any LLM to understand privacy aspects. Caution should be exercised when providing private, privileged, or protected data.

Ultimately, LLMs do not oversee prompts for integrity or consistency. For example, when asked to triage a patient described as both ambulatory and unconscious, ChatGPT may not identify the contradiction in that a patient cannot be both. Often, ChatGPT assigns a triage code without recognizing such discrepancies.

Determining the attitudes and beliefs of staff is critical for the successful introduction of new technologies in medical institutions. Failure to establish new technologies in this setting is not always due to the nature of the system but rather because employees (clinicians) have insufficient knowledge about the acceptability, acceptance, and adoption process [26]. This reinforces the importance of value cocreation between technology and clinical leadership.

Finally, the use of LLM for disaster triage would require considerable research for the development of a useable point-of-care tool. Clearly expecting providers to type text, including prompt and patient data, would be unworkable and a more ergonomically acceptable interface would be needed.

#### Research Implications

Although this study suggests that the current ChatGPT version 4 does not adequately triage simulated patients using START, it does not preclude that AI may be a useful tool for triage. This study used the commercial version of ChatGPT, which was not fine-tuned for triage. A specifically fine-tuned model may be more accurate. Furthermore, other AI and machine-related technologies—such as k-nearest neighbors, support vector machines, or neural networks—may perform better.

While prompt dependency was clearly shown in this study, further, more structured research into the systematic features of effective and ineffective prompts is clearly indicated.

Finally, this study demonstrates that gage R and R (a statistically validated study design) is a practical method that researchers can use to evaluate the repeatability, reproducibility, and accuracy of AI-guided tools. AI solutions should be thoroughly studied before implementation into clinical practice—with the same attention to statistical rigor as one would use before introducing a new drug or medical device.

### Conclusions

This study indicates that ChatGPT version 4 is insufficient to triage simulated disaster patients via the START protocol. It demonstrated suboptimal consistency, with its overall precision falling below 50%. The overall accuracy of triage was only 63.9%. Health care professionals are advised to exercise caution while using commercial LLMs for vital medical determinations, given that these tools may commonly produce inaccurate data, colloquially referred to as hallucinations or confabulations. AI-guided tools should undergo rigorous statistical evaluation—using methods such as gage R and R—before implementation into clinical settings.

## Appendix

Multimedia Appendix 1 Sample patient vignettes.

Multimedia Appendix 2 Prompts.

Multimedia Appendix 3 Python script.

## Abbreviations

AI: artificial intelligence
API: application program interface
AV: appraiser variation
LLM: large language model
START: Simple Triage and Rapid Treatment

## Data Availability

The data sets generated during or analyzed during this study are available from the corresponding author upon reasonable request.
