# Supplementary material for: Accuracy of a Commercial Large Language Model (ChatGPT) to Perform Disaster Triage of Simulated Patients Using the Simple Triage and Rapid Treatment (START) Protocol: Gage Repeatability and Reproducibility Study
Source: J Med Internet Res. 2024 Sep 30;26:e55648. doi: 10.2196/55648 (PMC11474136; doi:10.2196/55648)
Supplement: Multimedia Appendix 1 [file jmir_v26i1e55648_app1.pdf]

History: Victim of high-speed motor vehicle collision at multiple vehicle incident. Found in front seat of van with seat belt fastened. Unresponsive. Examination: Unresponsive. In front seat of van with seatbelt fastened. Large open chest wound. No signs of life. No respiration with jaw thrust. Pulse: 0. Respiratory rate 0. Blood Pressure: 0. GCS: 3. Unable to Ambulate.

History: Laceration to base of right thumb. No motor defects. No paresthesia. States normal movement. Denies any other injuries. Examination: Alert, oriented, no apparent distress. Pulse: 74. Respiratory rate 16. Blood Pressure: 155/40. GCS: 15. Ambulatory.

History: Highway speed MVC. Vehicle burst into flames. Extricated self through window. Wearing seatbelt. Airbag deployed. Complains of low back pain, pain in right ribs, and pain in left knee. Examination: Alert, oriented, no apparent distress. Pulse: 70. Respiratory rate 24. Blood Pressure: 152/75. GCS: 15. Unable to Ambulate
