# Supplementary material for: Accuracy of a Commercial Large Language Model (ChatGPT) to Perform Disaster Triage of Simulated Patients Using the Simple Triage and Rapid Treatment (START) Protocol: Gage Repeatability and Reproducibility Study
Source: J Med Internet Res. 2024 Sep 30;26:e55648. doi: 10.2196/55648 (PMC11474136; doi:10.2196/55648)
Supplement: Multimedia Appendix 2 [file jmir_v26i1e55648_app2.pdf]

Note: In all prompts, the $$$$ is replaced with the patient vignette.

PROMPT 1
Take the role of a trained paramedic. Strictly use the "START (Simple triage and rapid treatment)" algorithm. Do not deviate from the START algorithm. Do not use other triage methods. Pay attention to walking status, breathing status, respiratory rate, capillary refill time, presence of radial pulse, and ability to obey commands. Triage the following patient by assigning to one of the following four categories: Red, Yellow, Green, Black. $$$$ Respond only with the color code and no other text.

PROMPT 2
Take the role of a trained paramedic. Strictly use the "START (Simple triage and rapid treatment)" algorithm. Cross-check each patient with this website https://emtprep.com/resources/article/performing-start-triage detailing the START algorithm. Do not deviate from the START algorithm. Do not use other triage methods. Pay attention to walking status, breathing status, respiratory rate, capillary refill time, presence of radial pulse, and ability to obey commands. Triage the following patient by assigning to one of the following four categories: Red, Yellow, Green, or Black. $$$$ Answer only with the color code and no other text.

PROMPT 3
Using the website https://medictests.com/units/start-triage, triage the following patient:
$$$$ Answer only with the color code and no other text.

PROMPT 4
Input: $$$$. Output: using start triage algorithm triage this patient. Answer only with the color code and no other text.

PROMPT 5
Examine all known medical databases and then triage the following patient using the START protocol.
$$$$ Answer only with the color code and no other text.

PROMPT 6
Simple triage and rapid treatment (START) refers to the triage method developed in 1983 by the Newport Beach Fire Department and Hoag Hospital in California. START is a triage method used by first responders during a mass casualty incident (MCI) to quickly and efficiently classify victims based on the severity of their injuries.
Classifications

The goal of triage is to do the “greatest good for the greatest number” of victims. To achieve this goal, START uses four color-coded categories that victims are assigned after a quick evaluation:

Deceased or expected to die (Black)
Immediate (Red)
Delayed (Yellow)
Walking wounded/minor injuries (Green)

The colors correspond to triage tags, tape, or designated areas, although triage tags are the most commonly used in the field.

Sorting Victims

Upon arrival at the scene, the first step is to call out to victims and ask anyone who can hear your voice and walk to go to a designated location. These are the walking wounded and are considered green or third-priority patients.

The second step is focused on nonambulatory victims. Go to the nearest victim and assess respiratory status. If the patient is not breathing, open the airway. If they start breathing, they are given a red tag and are considered a priority patient. If they do not start breathing, they are given a black tag.

Any victim who is spontaneously breathing faster than 30 breaths/minute or fewer than 10 breaths/minute is given a red tag.

If a patient is breathing normally, check for bilateral radial pulses. If a pulse is absent, tag them as red. If both pulses are present, ask them to follow a simple command, such as holding up two fingers. If they can follow your command, they are tagged as yellow and in the delayed category. Move on to the next victim and repeat.

Triage the following patient using this START algorithm: $$$$

Answer only with the color code and no other text.

PROMPT 7

Triage the following patient using START:$$$$ Answer only with the color code and no other text.

PROMPT 8

This is a sample patient vignette: $$$$

Act as a prehospital provider at a disaster site. Use the information below to triage this patient.

Simple triage and rapid treatment (START) is a triage method used by first responders to quickly classify victims during a mass casualty incident (MCI) based on the severity of their injury. The method was developed in 1983 by the staff members of Hoag Hospital and Newport Beach Fire Department located in California, and is currently widely used in the United States.[1]

Classification

First responders using START evaluate victims and assign them to one of the following four categories:

- Deceased/expectant (black)
- Immediate (red)
- Delayed (yellow)
- Walking wounded/minor (green)

The colors correspond to triage tags, which are used by some agencies to indicate each victim's status, although physical tags are not necessary if patients can be physically sorted into different areas.

Responders arriving to the scene of a mass casualty incident may first ask that any victim who is able to walk relocate to a certain area, thereby identifying the ambulatory, or walking wounded, patients. Non-ambulatory patients are then assessed. The only medical intervention used prior to declaring a patient deceased is an attempt to open the airway. Any patient who is not breathing after this attempt is classified as deceased and given a black tag. No further interventions or therapies are attempted on deceased patients until all other patients have been treated. Patients who are breathing and have any of the following conditions are classified as immediate:

Respiratory rate greater than 30 per minute;
Radial pulse is absent, or capillary refill is over 2 seconds;
Unable to follow simple commands

All other patients are classified as delayed.

Answer only with the color code and no other text.

PROMPT 9
Using the START Triage protocol determine the best way to Triage the following patient: $$$$ Answer only with the color code and no other text.
