# Supplementary material for: Accuracy of a Commercial Large Language Model (ChatGPT) to Perform Disaster Triage of Simulated Patients Using the Simple Triage and Rapid Treatment (START) Protocol: Gage Repeatability and Reproducibility Study
Source: J Med Internet Res. 2024 Sep 30;26:e55648. doi: 10.2196/55648 (PMC11474136; doi:10.2196/55648)
Supplement: Multimedia Appendix 3 [file jmir_v26i1e55648_app3.pdf]

```python
import requests
import json
import re
import sys
import time
import numpy as np
import pandas as pd
import mysql.connector
import local_settings
from utils import start_to_nato

NUMBER_REPEATS = 10

endpoint = "https://api.openai.com/v1/chat/completions"

api_key = local_settings.GPT_API_KEY

# The request headers, including the API key for authentication
headers = {
    "Content-Type": "application/json",
    "Authorization": f"Bearer {api_key}"
}

cnx = mysql.connector.connect(user=local_settings.MYSQL_USER,
                              password=local_settings.MYSQL_PASS,
                              host=local_settings.MYSQL_HOST,
                              database=local_settings.MYSQL_DB)
cursor = cnx.cursor(dictionary=True)
cursor.execute("SELECT PatientNumber, PresentHistory, Ambulatory, ExamGeneral, Pulse, Resp, BP, GCS,
Triage_START FROM patient_data WHERE PatientNumber = 5000")
standards = cursor.fetchall()

cursor.execute("SELECT * FROM prompt")
prompts = cursor.fetchall()

df = pd.DataFrame(columns=['case_id', 'prompt_id', 'nato_standard', 'nato_assigned', 'message'])

for prompt in prompts:
    context = prompt['text']
    context_id = prompt['id']

    # All prompts must contain the $$$$ as which will be replaces by the case text
    context = context.replace("\\r", "")
    context = context.replace("\n", "")
    context = context.replace("\r", "")
    context_split = context.split("$$$$")
    context_before = context_split[0]
    context_after = context_split[1]

    for row in standards:
        case_id = row['PatientNumber']
        if row['Ambulatory'] > 0:
            ambulatory = "Ambulatory"
        else:
            ambulatory = "Unable to Ambulate"
        case_text = f"History: {row['PresentHistory']}. Examination: {row['ExamGeneral']}. Pulse:
{row['Pulse']}. Respiratory rate {row['Resp']}. Blood Pressure: {row['BP']}. GCS: {row['GCS']}.
{ambulatory}."

        start_standard = row['Triage_START']
        nato_standard = start_to_nato(start_standard)
        start_list =[]

        # split the context before and after the $$$$ and insert the case text
        content = f"{context_before}\n {case_text}\n {context_after}"


        # The request body, including the context and prompt
        data = {
            "model": "gpt-4",
            "messages": [{"role": "assistant",
                          "content": content
                          }]
```

```
        }

        for x in range(NUMBER_REPEATS):
            # Send a post request to the API endpoint with the request headers and body
            while True:
                time.sleep(1)
                response = requests.post(endpoint, headers=headers, data=json.dumps(data))

                # Check the response status code to make sure the request was successful
                if response.status_code == 200:
                    # Get the response text
                    response_text = response.text

                    # Load the response text as a JSON object
                    response_json = json.loads(response_text)

                    # Get the response text from the JSON object
                    response_message = response_json["choices"][0]["message"]["content"]

                    try:
                        response_message_cleaned = response_message.replace("\n", "")
                        response_message_cleaned = response_message_cleaned.replace(".", "")
                        response_nato = start_to_nato(response_message_cleaned)
                        if isinstance(response_nato, int):
                            if response_nato <= 4 and response_nato >= 1:
                                nato_assigned = response_nato
                            else:
                                nato_assigned = None
                        else:
                            nato_assigned = None

                    except IndexError:
                        response_message_cleaned = "Error in index"
                        nato_assigned = None

                    msg = (
                        f"For trial {x} of case {case_id} using prompt {context_id}  "
                        f"the standard was {nato_standard}. "
                        f"The response message was '{response_message_cleaned}' so NATO {nato_assigned}
was assigned."
                    )
                    print(msg)

                    try:
                        # Update the data frame and create a new row
                        df.loc[len(df.index)] = [case_id, context_id, nato_standard, nato_assigned,
response_message_cleaned]
                        start_list.append(nato_assigned)

                        # Update the mysql data
                        sql = f"INSERT INTO triage (case_id, prompt_id, nato_standard, nato_assigned,
message) VALUES ({case_id}, {context_id}, {nato_standard}, {nato_assigned},
'{response_message_cleaned}')"
                        cursor.execute(sql)
                        cnx.commit()
                    except Exception as error:
                          print("Exception:", type(error).__name__)

                    break

                else:
                    # If the request was not successful, print an error message
                    print("Failed to get response from API. Status code:", response.status_code)
                    break

        new_row = np.array(start_list)

print(df)
df.to_csv("startbot_results.csv", sep=',', encoding='utf-8')
cnx.close()
```
